# Clinical and diagnostic significance of apoptosis in the development of neutropenia and bacterial complications in newborns with respiratory distress syndrome

**DOI:** 10.1186/cc11720

**Published:** 2012-11-14

**Authors:** M Puchtinskaya

**Affiliations:** 1 Research Institute of Obstetrics and Pediatrics, Rostov-on-Don, Russia

## Background

The development of neutropenia with neonates with RDS on mechanical ventilation is a sign of the realization of bacterial complications [1,2]. The purpose of this study was the optimization of prevention of neonatal sepsis.

## Methods

With permission of the Ethics Committee 64 full-term newborns with RDS on mechanical ventilation, without clinical signs of infection, were retrospectively divided into two groups: with a decrease in absolute neutrophil count (M <2,000 cells/mm^3^) after 3 to 5 days of hospitalization (I, *n *= 30) and non-neutropenic (II, *n *= 34). The survey was conducted at admission and 3 to 5 days. They were studied on the content of lymphocytes in the early (AnnexinV-FITC^+^PI^-^) and late (AnnexinV-FITC^+^PI^+^) apoptosis by flow cytometry (AnnexinV^+^-labeled FITK and propidium iodide (PI^+^)-labeled PE; Saltag, USA), taking into account results on the cytometer (Beckman Coulter Epics XL, USA); the plasma level of granulocyte colony-stimulating factor (GCSF), fibroblast growth factor (FGF), anti-apoptosis soluble sFas-ligand (sFas-L) by ELISA (Victor, Finland; test system Cytimmune Sciences Inc., USA and Bender MedSystems GmbH, Austria). Points of cutoff were determined by ROC analysis. The statistical power of the study is 80% (α ≤ 0.05).

## Results

At admission, it was revealed that the patients in group I have a high content of lymphocytes in the early and late apoptosis, and low levels of GCSF, FGF, sFas-L in reference to group II (α ≤ 0.05). In group I, 27 patients at 3 to 5 days developed neutropenia (M <2,000 cells/mm^3^) and a reduction in plasma levels of GCSF, FGF, sFas-L and an increase in lymphocyte apoptosis (α ≤ 0.05). Sepsis developed in 22 children of group I and in only five patients of group II (α ≤ 0.05). The values of cutoff (sensitivity, specificity, accuracy) for prediction of neutropenia in neonates with RDS at admission to the ICU were: for GCSF - 1,556 pg/ml (85.1%, 75.6%, 79.6%, respectively); for sFAS-L - 5,870 pg/ml (69.56%, 74.4%, 71.9%); for FGF - 25.7 pg/ml (68%, 82%, 74.4%); for early apoptosis - 9.59% (82%, 93%, 74%); and for late apoptosis - 0.56% (94.1%, 98%, 85.3%).

## Conclusion

Reduction of proliferative factors (GCSF, FGF, sFas-L) and activation of apoptotic lymphocytes are markers of neutropenia and are associated with the high incidence of sepsis in infants with RDS.
